# How AI-Based Digital Rehabilitation Improves End-User Adherence: Rapid Review

**DOI:** 10.2196/69763

**Published:** 2025-08-14

**Authors:** Mahsa MohammadNamdar, Michael Lowery Wilson, Kari-Pekka Murtonen, Eeva Aartolahti, Michael Oduor, Katariina Korniloff

**Affiliations:** 1 Injury Epidemiology and Prevention (IEP) Research Group, Turku Brain Injury Centre, Department of Clinical Neurosciences, Turku University Hospital and University of Turku, Vähä Hämenkatu 2b, Turku, 20500, Finland, 358 29 450 5000; 2 Section for Oral Health, Heidelberg Institute of Global Health (HIGH), Heidelberg University, Heidelberg, Germany; 3 Institute of Rehabilitation, Jamk University of Applied Sciences, Jyväskylä, Finland

**Keywords:** artificial intelligence, AI, rehabilitation, digital rehabilitation, compliance, adherence, PRISMA, Preferred Reporting Items for Systematic Reviews and Meta-Analyses

## Abstract

**Background:**

The integration of artificial intelligence (AI) in rehabilitation technology is transforming traditional methods, focusing on personalization and improved outcomes. The growing area of AI in digital rehabilitation (DR) emphasizes the critical role of end-user compliance with rehabilitation programs. Analyzing how AI-driven DR tools can boost this compliance is vital for creating sustainable practices and tackling future challenges.

**Objective:**

This study seeks to assess how AI-based DR can improve the end-user compliance or adherence to rehabilitation.

**Methods:**

Following the updated recommendations for the Cochrane rapid review methods guidance and PRISMA (Preferred Reporting Items for Systematic Reviews and Meta-Analyses) guidelines, a systematic literature search strategy was led in PubMed, which yielded 922 records, resulting in 6 papers included in this study.

**Results:**

The reviewed studies identified 6 key ways in which AI enhances end-user compliance in rehabilitation. The most prevalent method (in 4 studies) involves motivating and engaging users through features like exercise tracking and motivational content. The second method, also noted in 4 studies, focuses on improving communication and information exchange between health care providers and users. Personalized solutions tailored to individual cognitive styles and attitudes were highlighted in 3 studies. Ease of use and system usability, affecting user acceptability, emerged in 2 studies. Additionally, daily notifications, alerts, and reminders were identified as strategies to promote compliance, also noted in 2 studies. While 5 studies looked at AI’s role in improving adherence, 1 study specifically assessed AI’s capability for objective compliance measurement, contrasting it with traditional subjective self-reports.

**Conclusions:**

Our results could be especially relevant and beneficial for rethinking rehabilitation practices and devising effective strategies for the integration of AI in the rehabilitation field, aimed at enhancing end-user adherence to the rehabilitation regimen.

## Introduction

Increased population growth in the global south, aging demographics in the global north, and rising numbers of individuals in both contexts with chronic conditions highlight the growing need for multidisciplinary rehabilitation worldwide. Rehabilitation is defined as “a set of interventions designed to optimize functioning and reduce disability in individuals with health conditions in interaction with their environment” [1]. Access to rehabilitation services plays a crucial role in providing comprehensive care and support for individuals in need. However, traditional rehabilitation services present significant limitations, including time constraints, long distances to travel, high costs, lack of human resources, and limited availability of clinical facilities, leaving a large unmet need and subsequent lack of participation [2,3].

The growing trend toward digitized health care, the use of portable technology, and events such as the COVID-19 pandemic have exacerbated the challenges that rehabilitation services face. The social distancing measures implemented during the COVID-19 pandemic provided a considerable push for the expanded use of digital rehabilitation (DR) modalities by numerous types of health care professionals [4]. DR has the potential to advance end-user (patient) and therapist collaboration to enhance the outcomes, improve performance challenges and quality of life, decrease health care costs, and tackle other key rehabilitation obstacles [5,6]. DR involves incorporating digital technologies into the rehabilitation process, which encompasses, but is not limited to telehealth and remote rehabilitation apps and services, a range of sensor and information and communication technologies, artificial intelligence (AI)–driven and robot-assisted tools, wearables, as well as email, video, speech, and SMS text messaging solutions [3,4].

The application of AI is revolutionizing almost all subfields of health care, including rehabilitation [7]. AI is described as the capacity of a machine to do a functional task under the intelligent supervision of humans [8]. AI tools are becoming more adept at learning from extensive and intricate data, using algorithms to acquire knowledge, analyze, and subsequently aid in different clinical and rehabilitation procedures [9].

Rehabilitation technology supported by AI signifies a groundbreaking and revolutionary method in the rehabilitation sector, which can be used to customize and complement the overall quality of traditional rehabilitation strategies [10]. AI has become a crucial component of DR, transforming and improving evaluation, screening, therapy, and monitoring through identifying patterns within vast quantities of health care data [10]. These patterns can then be used in designing tailored and personalized rehabilitation strategies and treatment regimens and comprehensive care planning [10-12]. AI tools support the automation of many rehabilitation tasks, injury prevention, prompt referral decisions, remote rehabilitation, monitoring and forecasting the end-user progress, and the creation of assistive technologies [12,13].

AI technologies provide efficient and enhanced rehabilitation access with optimized outcomes, which have the potential to improve client adherence and motivation [4,10,12]. Some of these technologies include AI-based digital and personalized rehabilitation mobile apps, AI-driven virtual reality and augmented reality rehabilitation, sensors, robotic devices, and AI-powered gamification and telerehabilitation [4,10].

Despite the key benefits of these technology-driven interventions—such as improved accessibility, affordability, and their availability from the convenience of homes—most of the AI-based rehabilitation applications are in the early stages of development [12,14-16]. However, the application of AI in redesigning rehabilitation to tackle upcoming challenges will intriguingly grow [4].

Compliance or adherence to long-term treatments, including rehabilitation, poses a significant health care challenge when managing chronic illnesses and is crucial for the success of rehabilitation and end-user recovery. While it signifies the end user’s readiness to engage in and dedicate themselves to the treatment and prescribed therapeutic routine, this commitment aims to prevent complications and the reoccurrence of the disease by enhancing daily activities, quality of life, and overall outcomes [17]. Although the terms compliance and adherence are different in some ways, the 2 words are used interchangeably by medical professionals [18]. Compliance or adherence guarantees that there is willing, cooperative, and engaged interaction between the client and the health care provider [19].

Several factors and explanations contribute to the complexity of compliance or adherence to rehabilitation. In medical and rehabilitation settings, adherence refers to how well individuals follow the clinical recommendations that have been mutually established [20]. In this study, we refer to rehabilitation adherence as the end user’s (patient’s) compliance or adherence to, engagement in, and acceptability of the rehabilitation program. Reasons for the end user not adhering to rehabilitation plans may include skepticism about treatment benefits, concerns about side effects, financial limitations, the complexity of the treatment plan, and insufficient support from family and peers [19,21]. Prior studies have indicated that patients who adhere more closely to treatment plans tend to experience improved outcomes [22]. However, there is an overall lack of evidence in the area of compliance or adherence, and in what ways AI can improve end-user rehabilitation compliance or adherence is unknown. AI in DR is an emerging field, and considering that end-user compliance or adherence to a rehabilitation regimen plays a significant role, comprehending how AI-based DR can impact the end-user rehabilitation compliance or adherence is crucial for establishing sustainable DR practices and meeting the challenges ahead. The aim of this rapid review (RR) lies in understanding the impact of AI-based DR tools on the improvement of end-user rehabilitation compliance or adherence. This review answers the following research question: “How can AI-based DR improve end-user compliance or adherence to rehabilitation?”

## Methods

### Study Design

A Cochrane RR is “a form of knowledge synthesis that accelerates the process of conducting a traditional systematic review through streamlining or omitting specific methods to produce evidence for stakeholders in a resource-efficient manner” [23]. It primarily provides prompt evidence for decision-making regarding urgent and high-priority health issues [23].

RR is a type of systematic review that aims to balance time limitations with the need to address bias [24]. However, RRs may not include all elements of a systematic review, with differences in the dimensions of time, resources, and searches. For example, Cochrane RRs should take no longer than 6 months; they may be less comprehensive, and a small number of databases are selected for searches; they may exclude hand-searching and gray literature; and some limits such as years and language may be applied in RRs [24,25].

This review is in compliance with the updated recommendations for the Cochrane RR methods guidance and the PRISMA (Preferred Reporting Items for Systematic Reviews and Meta-Analyses) recommendations (Checklist 1) [24,26]. The Cochrane RR guidance consists of 24 specific recommendations supporting the conduct of RRs to enhance the efficiency of the review process [24].

### Literature Search

PubMed was searched in May 2024, and a combination of MeSH terms and keywords on the following themes was used: AI, DR, and end-user compliance or adherence. A copy of the PubMed search strategy is included as Multimedia Appendix 1.

### Study Selection

Titles and abstracts were initially screened through applying the eligibility criteria by 2 reviewers (MM and MLW) independently. The only software that was used to facilitate this process was Microsoft Excel to aid in the display and tabulation of the information. For retained papers, full texts were obtained, and again, a subset of full texts was screened by 2 researchers (MM and MLW). Differences and discrepancies were discussed, and dual screening was continued until convergence was reached.

Studies were included when they encompassed the following elements in line with the population, intervention, comparison, and outcome framework for evidence-based practice:

- Population: Adults (≥18 years of age) undergoing formal rehabilitation.
- Intervention: Any type of AI-based applications and tools, used by end users or health care providers, in inpatient, outpatient, or community-based settings.
- Comparison: For interventional studies, the control group is defined as those not receiving AI-supported rehabilitation.
- Outcome: End-user rehabilitation compliance or adherence.
- Study types: Any study design.
- Other: English language studies published from January 1, 2012, onward.

Protocols, books, theses, editorials, conference abstracts, and non-English language studies were excluded. Studies focusing on psychiatric rehabilitation were intentionally excluded since distance- and digital-based mental health interventions have been widely available, even before the internet age with counseling being done via telephone. Studies focusing on robotics and AI were also excluded, as this is a sufficiently large field that would require a separate review. Considering the recent use of AI applications in the health care field and a surge of advancements in AI in 2012, it was deemed not necessary to review papers published before 2012.

### Data Extraction and Management

Data extraction was conducted by a single researcher and then verified for consistency, accuracy, and completeness by a second independent researcher. It included population characteristics, rehabilitation field and setting, intervention details including AI subset or algorithm, purpose of the device and the technology used, the study aims and its characteristics evidencing drawbacks and advantages reported by respective authors, major findings, and the way AI impacts the end-user compliance or adherence.

### Quality Assessment

The Cochrane Risk of Bias tool 2.0 [27] was used for randomized controlled trials (RCTs) to assess the quality of studies. The tool inquires about various aspects in 5 domains where bias could potentially arise. The five domains include (1) bias from randomization, (2) bias from deviations in interventions, (3) bias from missing outcome data, (4) bias in outcome measurement, and (5) bias in reported result selection.

### Data Synthesis

Due to the diversity in study designs and outcome measures, the results were synthesized in a narrative format.

## Results

### Overview

**Figure 1.**
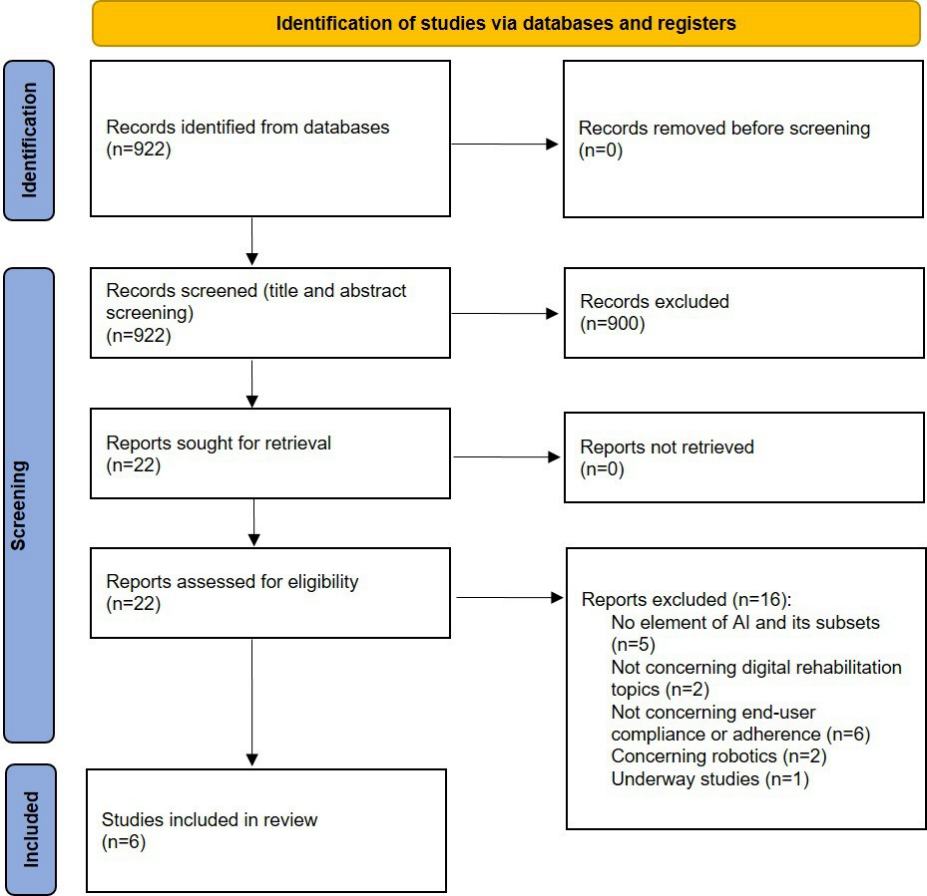
PRISMA (Preferred Reporting Items for Systematic Reviews and Meta-Analyses) flowchart of the results from the literature search. AI: artificial intelligence.

In the initial paper search, 922 unique papers were identified. After screening, 6 papers met the eligibility criteria and were included (Figure 1).

### Study Characteristics and Interventions

Characteristics of the included studies and their relevant interventions are presented in Table 1. Of the 6 publications, 3 were RCTs [28-30], 2 were pilot studies [31,32], and 1 was a proof-of-concept study [33]. The number of participants recruited into the included studies ranged from 6 to 500 participants. Studies were conducted in Canada (n=1), Israel (n=1), the Republic of Korea (n=1), Thailand (n=1), Italy (n=1), and the United States (n=1).

**Table 1.** Characteristics of the included studies and their interventions.

Reference (year, country)	Study design	Rehabilitation field and setting	Sample characteristics	Intervention
Chae et al (2020) [29] (Republic of Korea)	RCT^a^	Upper limb rehabilitation, home-based	End users with chronic stroke, intervention: n=17; mean age 58.3 (SD 9.3) years; control: n=6, mean age 64.5 (SD 9.6) years	Performing the home rehabilitation exercises while using wearable sensors embedded in a commercial smartwatch
Aharon et al (2022) [28] (Israel)	RCT	Cardiac rehabilitation, clinic-based	End users discharged from a cardiovascular event, intervention: n=95; mean age 61.3 (SD 14.6) years; control: n=500; mean age 65.4 (SD 10.4) years	Using the Well-Beat system, ongoing personalized text messages for each end user were generated to create continuity and reinforce desired behaviors
Burns et al (2018) [33] (Canada)	Proof of concept	Shoulder physiotherapy, home-based	Healthy adult participants, n=20, mean age 28.9 years; range 19‐56 years	Performing 20 repetitions of 7 shoulder exercises bilaterally while using a smartwatch device with embedded inertial sensors
Thiengwittayaporn et al (2023) [30] (Thailand)	RCT	Physical therapy, home-based	End users with primary knee osteoarthritis, intervention: n=42; mean age 62.2 (SD 6.8) years; control: n=40; mean age 63.0 (SD 9.7) years	Receiving information regarding knee osteoarthritis disease background and exercise instruction through mobile app self-directed exercise guidance
Capecci et al (2023) [31](Italy)	Prospective, pre- or postintervention pilot study	Motor and respiratory rehabilitation, home-based	Participants with post–COVID-19 condition and Parkinson disease, n=21; mean age 61.1 (SD 10.5) years	Using ARC Intellicare, a telerehabilitation solution based on the use of multiple wearable sensors, a mobile device, and algorithms of AI^b^
Ramkumar et al (2019) [32] (United States)	Pilot study	Total knee arthroplasty, home-based	End users undergoing primary total knee arthroplasty, n=25; mean age 64.3 years	Performing daily exercises and a weekly survey while using a knee sleeve paired with the end user’s iPhone and a mobile app

a RCT: randomized controlled trial.

b AI: artificial intelligence.

There were diverse rehabilitation settings, and most were home-based. Only in 1 study was the rehabilitation in a clinical setting. The treated conditions included stroke, cardiovascular disease, osteoarthritis, post–COVID-19 condition, and Parkinson disease. While 4 studies used wearable sensors as an intervention, in 1 study, end users received information regarding the disease background and exercise instruction through a mobile app, and in the other study, ongoing personalized SMS text messages for each end user were generated to create continuity and reinforce desired behaviors.

### Outcomes and Results Summary

Outcome measures and results summary of the included studies are presented in Table 2. The outcomes measured in the included studies fell into 3 categories: feasibility, accuracy, and usability of the rehabilitation system; end-user compliance or adherence, engagement, and acceptability; and clinical outcomes (eg, functional improvements, range of motion, and quality of life). However, our focus is on the second category of outcomes.

**Table 2.** Outcomes and results summary of the included studies.

Reference	Outcome measures		Results summary
Primary measures (end-user compliance or adherence)	Other measures (feasibility, accuracy, and usability or clinical outcomes)
Chae et al [29]	Dropout rate and home exercise performance average time as measures for the end-user compliance or adherence	Accuracy of the developed rehabilitation system, functional assessment test, shoulder range of motion	A decreased dropout rate, increased home exercise performance average time, a significant increase in functional recovery and shoulder flexion
Aharon et al [28]	End-user rehabilitation adherence	None	Significant improvement in end-user adherence and participation in the rehabilitation program
Burns et al [33]	The frequency and duration an end user is engaged with exercises as measures for the end-user compliance or adherence	Classifier performance in the measurement of frequency and duration of an end-user engagement in prescribed exercises	Feasibility of robust and highly accurate classification of sensor data in the measurement of frequency and duration of an end-user engagement in prescribed exercises
Thiengwittayaporn et al [30]	Ability to correctly perform the rehabilitation exercises as measures for the end-user compliance or adherence	Range of motion, Knee Injury and Osteoarthritis Outcome Score, and Knee Society Score	Improved accuracy of rehabilitation exercises, as measures for the end-user adherence, stronger overall functional outcomes, better daily life, ability to do sports and recreation, higher satisfaction and expectation, and a significantly better quality of life
Capecci et al [31]	Adherence to the rehabilitation program	Safety, usability, and acceptability of the rehabilitation program, clinical effectiveness (disability in basal activity of daily living, respiratory outcomes, endurance and fatigue, mood, and quality of life)	Improved end users’ adherence, improved system usability scale score, clinical outcome measures, and no side-effect reports
Ramkumar et al [32]	Rehabilitation compliance	Feasibility validation, mobility, knee range of motion, patient-reported outcome measures, opioid use	Improved rehabilitation compliance, the provision of a continuous stream of data without any loss, improved mobility and patient-reported outcome measures, and stopped opioid use

### Risk of Bias Summary

Three of the studies [28-30], which were RCTs, were assessed using the Cochrane risk of bias tool [27]. One of the studies had an overall risk of bias of “some concerns” [30], and 2 studies had an overall high risk of bias [28,29]. In total, 1 study had a high risk of bias arising from the randomization process [29], all 3 studies had a risk of bias of some concerns in the domain of bias due to deviations from intended interventions, and in the domain of bias in measurement of the outcome, 1 study had a high risk of bias [28], and 2 studies had a risk of bias of some concerns [29,30].

### The Role of AI

Summarized information regarding the system and technology used by interventions in the included studies and the role of the used AI subset is presented in Table 3.

**Table 3.** Characteristics of the systems used in interventions: technology type, artificial intelligence (AI) subset, and role.

Reference	AI subset	System and technology type	Purpose or role of the AI-based device or system
Chae et al [29]	Machine learning (ML) algorithm implemented by a convolutional neural network ascertaining what types of sensor data can detect home exercise activities most accurately via a cross-validation test	Activity monitoring using wearables (smartwatch, smartphone, and apps [Android Studio 2.3, Google])	The AI-based rehabilitation system connected end users and therapists at a distance and made it possible to share end users’ home exercise data with therapists at remote locations. The system assisted participants in the intervention group to record their exercise time, obtain their own home exercise results, and communicate with a clinician, yielding visible improvement and acting as a motivation—features that were not available to those in the control group.
Aharon et al [28]	A real-time ML algorithm processing the digital questionnaires	Well-Beat platform, which created a profile for each end user by processing a digital questionnaire assessing end users’ initial state	After processing the questionnaires, the system created a profile for each end user and presented it on the health care providers’ toolbar. The toolbar included the end user’s persistence level, readiness for change level (maturity), self-efficacy level, main motivational driver, and barrier, as well as what the health care provider should watch out for in communication with them. Based on this information, the engine recommended the personalized end-user dialogue to use, in terms of the tone, the style, and motivation factors, as well as what to avoid saying.
Burns et al [33]	Supervised ML approach	Exercise recognition using wearables, that is, smartwatch device with embedded inertial sensors (Apple Watch [series 2 and 3] with the PowerSense app, sampling at fs=50 Hz)	The AI-based system assessed the feasibility of performing shoulder physiotherapy exercise recognition with inertial sensor data recorded from a wrist-worn device to enable objective measurement of home shoulder physiotherapy adherence.
Thiengwittayaporn et al [30]	Rule-based and AI techniques (eg, determining the disease stage based on decision tree)	Education and assessment for the stage of disease via Love-Your-Knee mobile app (Android)	The AI-based system was used as a personalized solution and recommending the appropriate exercise types and the number of sets for each end user by assessing the stage of the disease, monitoring disease progression, and promoting physical therapy and rehabilitation exercise.
Capecci et al [31]	Algorithms of AI (patent pending), a neural network was used to recognize the performed exercise and segment the data into single repetitions	The ARC Intellicare system (an AI-powered and inertial motion unit-based mobile platform) consisting of a set of 5 inertial sensor inserted in slap supports, a tablet with a dedicated app, and a charging station	The AI-based system allowed rehabilitation professionals to prescribe exercises according to specific therapeutic needs and to monitor end users’ performances and progresses remotely. The counting of the number of exercise repetitions correctly performed was the output of the developed AI algorithm. Real-time feedback was provided to the end user through the app user interface.
Ramkumar et al [32]	ML algorithms	Activity monitoring using wearables (knee sleeve, personal iPhones [Apple], and mobile app termed TKR [Focus Ventures])	The system transmitted the wearable knee sleeve motion data to the smartphone, then transmitted these and all other data to the dashboard, then analyzed these data by the ML algorithms to actively record and check daily compliance in order to provide automated reminder notifications based on the end user’s compliance to the program.

### The Ways AI Impacts End-User Rehabilitation Compliance or Adherence

In the included studies, the role of AI in rehabilitation and how it impacts end-user compliance or adherence to rehabilitation fell into 6 categories. End-user rehabilitation compliance or adherence improvement through motivation, engagement, and encouragement of end users was the first more cited procedure that was provided by AI-based rehabilitation systems or tools in the studies [28-30,32]. This was done, for example, by exercise tracking, using interactive features, and sending motivational content to end users to keep them engaged and to motivate them to adopt more desirable behaviors.

The second most cited approach was the enhancement of end-user rehabilitation compliance or adherence by facilitating information exchange and improving health care provider-end-user interaction and communication [28-31]. Third, offering a personalized end user–tailored solution was the other strategy supported by AI-based rehabilitation systems that could influence end-user compliance or adherence to rehabilitation [28,30,31], for example, by way of identifying end users’ cognition, coping styles, and attitudes. The fourth category was ease of use and simple system usability influencing end-user acceptability [31,32]*.* Providing daily notifications, alerts, and reminders for end users, as end users, was another procedure that AI-based rehabilitation systems used to improve end-user compliance or adherence to rehabilitation [28,32].

While 5 studies addressed the role of AI in end-user rehabilitation compliance or adherence improvement, 1 study evaluated the capacity of AI-based rehabilitation systems in objective measurement of end-user compliance or adherence [33]. Since adherence is typically assessed using subjective methods like relying on end users’ self-reports for home exercises, which may lack validity due to recall bias, social desirability, and misinterpretation, AI-based solutions could more easily and objectively monitor and assess the at-home adherence of exercise protocols by measuring the frequency and duration an end user is engaged with each of their prescribed exercises.

### Implications and Drawbacks

Table 4 presents summarized information on the implications for end-user rehabilitation compliance or adherence, along with the drawbacks and limitations reported within the included studies themselves.

**Table 4.** Summary of the included studies’ implications and reported drawbacks.

Reference	Implications concerning end-user rehabilitation compliance or adherence	Study drawbacks and limitations
Aharon et al [28]	A dropout rate could decrease, and home exercise performance average time could increase due to using an AI^a^-based rehabilitation device, which could be considered as measures for improved end-user compliance or adherence to rehabilitation	Small number of participants, discrepancy in the number of case and control participants, and the probability of selection bias
Chae et al [29]	To increase adherence, any intervention should be tailored to the end user’s preferences and behavioral profile, and this end user–tailored intervention approach could improve adherence with no change to the therapeutic regime	The potential bias related to the unblinded nature of this study with health care providers, the possible influence of the method of recruiting participants on the results
Thiengwittayaporn et al [30]	AI-based algorithms could be used to objectively monitor and assess the end-user rehabilitation compliance or adherence by measuring the frequency and duration an end user is engaged with exercises	The limited number of participants, no participants had symptomatic shoulder disorders, significantly younger age of the participants, and how well the proposed AI-based system would generalize to adherence monitoring in a clinical population is uncertain
Capecci et al [31]	An end user’s ability to accurately perform the exercises at the final follow-up reflects adherence to home exercise, which could be improved due to the role of AI-based rehabilitation regimen	The end users included in this study were able to efficiently use smartphones, which may not be reflective of a larger older adult population; according to the structure of the study, it was impossible to blind the participants to their intervention, which could potentially skew self-reported outcomes, and the majority of the end users were female participants
Ramkumar et al [32]	AI-based devices allow for real-time monitoring of several aspects of adherence: both daily adherence and repetition, based on exercise recognition	The small sample size and the absence of a control group
Burns et al [33]	The AI-based remote end-user monitoring system could offer the newfound ability to more completely evaluate the end-user rehabilitation compliance	The data represented a small cohort with no broadly generalizable conclusions, a small sample size, and the potential risk for selection and recall bias by end users

a AI: artificial intelligence.

## Discussion

### Principal Findings and Interpretation

Achieving end-user autonomy represents an important objective of rehabilitation in response to the growing population, aging demographics, and higher prevalence of chronic illnesses. The integration of AI in rehabilitation technology represents an innovative and transformative approach, offering the potential to enhance and tailor traditional rehabilitation methods also for improved overall effectiveness [10]. However, adhering to extended treatment regimens, such as what rehabilitation may often require, presents a significant health care challenge in the management of chronic conditions and is essential for the effectiveness of rehabilitation and end-user recuperation. This review showcases how technology-driven interventions affect end-user (patient) rehabilitation adherence and focuses on the use of AI in enhancing end-user rehabilitation compliance or adherence by providing a general summary of the topic. This study categorizes literature according to the combination of procedures that AI impacts end-user rehabilitation compliance or adherence to support the future challenge of implementing sustainable DR practices to address upcoming issues.

According to the findings, 6 distinct areas were found to be important in assessing the impact of AI on end-user rehabilitation compliance or adherence across the independent studies [28-33]. One method that was frequently mentioned in the studies for enhancing end-user compliance or adherence to rehabilitation involved using AI-based rehabilitation systems and tools to motivate, engage, and encourage end users [28-30,32]. Motivation is a significant personal factor that strongly affects adherence to rehabilitation [32], which can be supported by AI-based technologies. Due to the significance of establishing habits and nurturing internal motivation, AI-based rehabilitation systems could potentially promote consistency and strengthen end users’ desired behaviors by exercise tracking, using interactive features, music, video instruction, alerts, and awards, and sending motivational content to end users to keep them engaged and motivated [30]. Based on the findings, various aspects of AI-driven rehabilitation could enhance end-user engagement, such as real-time feedback and self-assessment features, and showcase daily progress [32].

Promoting information sharing and enhancing communication and interaction between health care providers and end users were other specific areas that AI-based rehabilitation systems could play a role in improving end-user rehabilitation compliance or adherence [28-31]. Numerous authors believe that developing a strong therapeutic bond with end users and effective health care provider-end-user communication greatly influences end-user adherence [34,35]. Communication-driven features of the AI-based rehabilitation tools and services could potentially strengthen the health care provider-end-user interaction and enhance communication. This was achieved by identifying the most effective communication approach for end users, tailoring personalized communication, and inspiring them to take action [28]. End users could benefit from such interventions by conveniently retrieving relevant medical information offered by these tools [30]. By facilitating the information exchange between the end user and the health care provider, the AI-powered rehabilitation tools and services can potentially support remote interventions. This represents a significant advancement in delivering services to vulnerable end users who must refrain from visiting health care centers during events like the COVID-19 pandemic [28].

Offering personalized solutions tailored to individual end users was a strategy supported by AI-driven rehabilitation systems that could impact end-user compliance or adherence to rehabilitation [28,30,31]. End users’ characteristics, values, and the variability among their requirements, lifestyle, habits, beliefs, and persistence should be taken into consideration in the development and implementation of interventions aimed at improving health [36,37]. Accordingly, in order to improve end-user adherence to rehabilitation protocols, interventions should be customized to align with the end user’s preferences and behavioral characteristics. Tailoring an AI-based rehabilitation intervention for each end user was determined by a comprehensive assessment of their medical records and profile, including aspects such as personality, coping mechanisms, willingness to change, and other factors, in addition to their behavior. This was achieved through, for example, targeted screening to identify end users’ cognitive frameworks, their attitudes and perspectives on the circumstances, the factors that inspire them, and the obstacles they face toward progress [28]. In the study of Capecci et al [31], AI algorithms allowed rehabilitation professionals to prescribe exercises according to end users’ specific therapeutic needs and to monitor their performances and progresses remotely, which led to real-time monitoring of several aspects of adherence and improved end-user compliance or adherence to rehabilitation. Similarly, Thiengwittayaporn et al [30] used AI techniques as a personalized solution for assessing the stage of the disease for each end user, monitoring disease progression, and promoting physical therapy and rehabilitation exercise. Therefore, rather than aiming for a one-size-fits-all optimal intervention, AI-based rehabilitation services help health providers identify the most suitable intervention for each individual end user at a specific moment. What may be highly effective for one end user can be ineffective, or even detrimental, for another. Additionally, the same intervention that initially shows promise in the rehabilitation process may prove inadequate, as the end user’s performance progresses.

Another category, which could be instrumental for the improvement of end-user rehabilitation compliance or adherence, was the usability and ease of use of the AI-based rehabilitation intervention [31,32]. Low usability, in terms of ease of use and suitability, and inadequate user-friendliness are primary factors leading to the neglect of technological systems, impacting individuals’ willingness to accept digital solutions and adherence to the treatment [38,39]. With their availability from the convenience of homes and being readily usable, AI-based rehabilitation tools and services could potentially contribute to end-user engagement and satisfaction and consequently rehabilitation compliance or adherence [32]. Moreover, AI technologies are commonly being integrated into smartphone apps, offering a variety of health care services including rehabilitation. The availability and ubiquity of smartphones, easy-to-use experience of the mobile apps, and platforms that do not necessitate any extra hardware beyond an end user’s own smart device have unleashed the possibilities of AI-driven rehabilitation tools and services to enhance end-user compliance or adherence to rehabilitation [32].

Additionally, the findings of this review suggest that the capability of AI-based tools and services in providing automated daily notifications, alerts, and reminders for end users had an impact on the improvement of end-user rehabilitation compliance or adherence [28,32]. Numerous studies have demonstrated the effectiveness of reminders, notifications, and alerts in improving health outcomes [40,41]. The AI-driven rehabilitation tools and services have the ability to remotely monitor end users’ progress and compliance with rehabilitation protocols, offering automated daily notifications, alerts, and reminders for end users to enhance end-user adherence to the program. For example, in the study of Ramkumar et al [32], the AI-based system actively recorded end users’ weekly data for the daily compliance check and provided automated reminder notifications whenever required, and the end users mentioned these notifications as reasons for their engagement with the system.

Another key finding of this review is the ability of AI-driven rehabilitation systems to accurately measure end-user rehabilitation adherence in an objective manner [33]. At present, there is a scarcity of tools and a lack of agreement on a standardized approach for accurately and objectively measuring end-user rehabilitation compliance or adherence, specifically in a home environment [33,42,43]. End-user rehabilitation compliance or adherence evaluated through subjective approaches, such as end users’ self-reports for home exercises, may lack validity. A less unexplored alternative is to assess the effectiveness and functionalities of AI-driven rehabilitation tools and services, offering the potential to use these technologies for objective end-user rehabilitation adherence measurement. AI-powered devices have the potential to enable the real-time tracking of various aspects of end-user rehabilitation compliance or adherence, including daily compliance and repetition, through exercise recognition [31]. In the study of Ramkumar et al [32], the AI-powered remote end-user monitoring system could provide a novel capability to assess end-user adherence to rehabilitation using machine learning algorithms. However, studies have taken various approaches to the evaluation of end-user rehabilitation adherence. For example, in the study of Chae et al [29], home exercise performance average time and a decrease in dropout rate were considered as measures for improved end-user adherence to rehabilitation. Burns et al [33] suggest that AI-based solutions could more easily and objectively monitor and assess the at-home adherence of exercise protocols by measuring the frequency and duration an end user is engaged with each of their prescribed exercises. Thiengwittayaporn et al [30] believe that an end user’s accurate performance in executing the exercises indicates adherence to home exercise; nevertheless, this may not consistently hold true.

The application of AI in end-user rehabilitation adherence is a relatively new and evolving field of study, and this topic is still in its infancy. Nevertheless, it is evident that certain features offered by AI-based rehabilitation can contribute to enhancing end-user compliance and adherence to rehabilitation protocols. Our aim was to offer a summary of the current role of AI in assessing and enhancing end-user adherence to rehabilitation programs by examining the ways by which AI can impact end-user adherence in rehabilitation. In general, the present findings indicate that AI could contribute to the improvement of end-user rehabilitation adherence by its capacity in motivating and engaging end users, facilitating information exchange and improving communication, offering personalized end user–tailored solutions, delivering convenience and ease of system use, providing automated daily notifications, alerts, and reminders for end users, and measuring end-user rehabilitation compliance or adherence with an objective and accurate approach.

### Strengths and Limitations

The evidence considered in this review was sourced from publications released within a 7-year time frame from the date of this RR, thereby enhancing the importance of the findings. Notwithstanding the methodological issues, all of the studies included in the analysis were published in peer-reviewed journals. However, it is important to address a few limitations of this review. First, we refrained from conducting a systematic literature review due to the limited number of studies available on the role of AI in measuring or enhancing end-user rehabilitation compliance or adherence. Moreover, due to the wide variety of study designs, it was deemed more suitable to perform an RR with synthesized results in a narrative format to present a comprehensive summary of the literature. Finally, the potential bias related to the unblinded nature of most of the included studies and the relatively limited number of participants complicates the definitive conclusions regarding the impact of AI-based DR on the end-user rehabilitation compliance or adherence.

### Future Directions

A deeper comprehension of the effectiveness of AI-based approaches in DR is required to support the improvement and objective measurement of end-user rehabilitation compliance or adherence. Future work should concentrate on implementing AI-based DR in the real world and relevant operational settings and evaluate the clinical significance of the intervention through RCTs comparing the impacts on particular demographic groups of these innovative solutions as opposed to conventional rehabilitation methods. Additionally, further research is needed to validate the use of specific AI models and use the AI’s complete capacity in the improvement of end-user rehabilitation compliance or adherence to achieve constructive change in population health, policy, and genuine medical revolution.

### Conclusions

This RR offers an insight into the role of AI in the DR and the approaches that AI uses to improve and measure the end-user rehabilitation compliance or adherence. Overall, the current findings suggest that AI-based rehabilitation can contribute to improved end-user compliance and adherence to rehabilitation programs by its capacity in motivating and engaging end users, facilitating information exchange and improving communication, offering personalized end user–tailored solutions, delivering convenience and ease of system use, providing automated daily notifications, alerts, and reminders for end users, and the objective measurement of end-user rehabilitation compliance or adherence. Our findings may be of particular interest and valuable in redesigning rehabilitation and strategizing the optimal integration of AI into the rehabilitation sector to address future challenges.

## Supplementary material

10.2196/69763 Multimedia Appendix 1 PubMed search strategy used for literature review.

10.2196/69763 Checklist 1 PRISMA (Preferred Reporting Items for Systematic Reviews and Meta-Analyses) checklist.

## Abbreviations

AI: artificial intelligence
DR: digital rehabilitation
PRISMA: Preferred Reporting Items for Systematic Reviews and Meta-Analyses
RCT: randomized controlled trial
RR: rapid review
