# Supplementary material for: How AI-Based Digital Rehabilitation Improves End-User Adherence: Rapid Review
Source: JMIR Rehabil Assist Technol. 2025 Aug 14;12:e69763. doi: 10.2196/69763 (PMC12352703; doi:10.2196/69763)
Supplement: Multimedia Appendix 1 [file rehab-v12-e69763-s001.docx]

Supplementary Table 1. PubMed Search Strategy Used for Literature Review

| Search number | Query | Results |
| --- | --- | --- |
| 1 | Rehabilitation OR "Rehab*" OR "Rehabilitation"[Mesh] OR "rehabilitation" [Subheading] OR "Rehabilitation, Vocational"[Mesh] OR "Rehabilitation of Speech and Language Disorders"[Mesh] OR "Rehabilitation Centers"[Mesh] OR "Physical and Rehabilitation Medicine"[Mesh] OR "Mouth Rehabilitation"[Mesh] OR "Cardiac Rehabilitation"[Mesh] OR "Stroke Rehabilitation"[Mesh] OR "Neurological Rehabilitation"[Mesh] OR "Correction of Hearing Impairment"[Mesh] OR "Exercise Therapy"[Mesh] OR "Substance Abuse Treatment Centers"[Mesh] OR "physical therap*" OR physiotherap* OR "Physical Therapy Modalities"[Mesh] OR "Physical Therapy Specialty"[Mesh] OR "Exercise" OR "Exercise therapy" OR "Exercise"[Mesh] OR "Exercise Movement Techniques"[Mesh] OR "Exercise Therapy"[Mesh] OR "Recovery of Function" OR "Recovery of Function"[Mesh] OR "occupational rehabilitation" OR "Occupational Therapy"[Mesh] | 1,257,678 |
| 2 | Digital OR "Digital Technolog*" OR "Digital Technology"[Mesh] OR "Digital Health"[Mesh] OR "Computers"[Mesh] OR "Computers, Handheld"[Mesh] OR "Signal Processing, Computer-Assisted"[Mesh] OR "Telemedicine" OR "Tele-medicine" OR "Telemedicine"[Mesh] OR "Telerehabilitation" OR "Tele-rehabilitation" OR "Telerehabilitation"[Mesh] OR "Telehealth" OR "Tele-health" OR "Internet-Based Intervention" OR "Internet-Based Intervention"[Mesh] OR "ehealth*" OR "e-health*" OR "mhealth" OR "m-health" OR "mobile health" OR "Mobile Health Units"[Mesh] OR "Virtual Reality" OR "virtual environment" OR "Virtual Exposure Therapy" OR "Virtual Reality"[Mesh] OR "Virtual Reality Exposure Therapy"[Mesh] OR "Gamifi*" OR "Exergam*" OR "Video Game*" OR "Exergaming"[Mesh] OR "Gamification"[Mesh] OR "Video Games"[Mesh] OR "Augmented Reality" OR "Augmented Reality"[Mesh] OR "Internet" OR "Internet"[Mesh] OR "Internet Use"[Mesh] OR "Internet of Things"[Mesh] OR "Internet Access"[Mesh] OR "Cell Phone" OR "Cell Phone"[Mesh] OR "Cell Phone Use"[Mesh] OR "Smartphone" OR "Mobile App*" OR "Mobile Applications"[Mesh] OR "Smartphone"[Mesh] OR "Telecommunication*" OR "Tele-communication*" OR "Telecommunications"[Mesh] OR "Communication Aids for Disabled"[Mesh] OR "Computer Communication Networks"[Mesh] OR "Satellite Communications"[Mesh] | 1,109,395 |
| 3 | "Artificial Intelligence" OR "AI" OR "Artificial Intelligence"[Mesh] OR "Machine Learning" OR "Machine Learning"[Mesh] OR "Unsupervised Machine Learning"[Mesh] OR "Supervised Machine Learning"[Mesh] OR "Neural Networks" OR "Neural Networks, Computer"[Mesh] OR "Deep learning" OR "Deep Learning"[Mesh] | 759,523 |
| 4 | "Treatment Adherence" OR "Treatment Compliance" OR "Treatment Adherence and Compliance"[Mesh] OR "Medication Adherence"[Mesh] OR "Patient Compliance"[Mesh] OR "Health Care Quality, Access, and Evaluation"[Mesh] OR "Access to Primary Care"[Mesh] OR "Health Services Accessibility"[Mesh] OR "Patient* autonomy" OR "Patient Self-Determination Act"[Mesh] OR "Personal Autonomy"[Mesh] | 8,202,345 |
| 5 | #1 AND #2 AND #3 AND #4 | 1,107 |
| 6 | #1 AND #2 AND #3 AND #4 (Filters: English, from 2012 - 2024) | 922 |
