# Supplementary material for: How AI-Based Digital Rehabilitation Improves End-User Adherence: Rapid Review
Source: JMIR Rehabil Assist Technol. 2025 Aug 14;12:e69763. doi: 10.2196/69763 (PMC12352703; doi:10.2196/69763)
Supplement: Checklist 1 [file rehab-v12-e69763-s002.docx]

| **Section and Topic** | **Item #** | **Checklist item** | **Location where item is reported** |
| --- | --- | --- | --- |
| **TITLE** | | |  |
| Title | 1 | Identify the report as a systematic review. | Page 1 |
| **ABSTRACT** | | |  |
| Abstract | 2 | See the PRISMA 2020 for Abstracts checklist. | Page 1 |
| **INTRODUCTION** | | |  |
| Rationale | 3 | Describe the rationale for the review in the context of existing knowledge. | Page 2 |
| Objectives | 4 | Provide an explicit statement of the objective(s) or question(s) the review addresses. | Page 3 |
| **METHODS** | | |  |
| Eligibility criteria | 5 | Specify the inclusion and exclusion criteria for the review and how studies were grouped for the syntheses. | Page 4 |
| Information sources | 6 | Specify all databases, registers, websites, organisations, reference lists and other sources searched or consulted to identify studies. Specify the date when each source was last searched or consulted. | Page 4 |
| Search strategy | 7 | Present the full search strategies for all databases, registers and websites, including any filters and limits used. | Included in the supplements. |
| Selection process | 8 | Specify the methods used to decide whether a study met the inclusion criteria of the review, including how many reviewers screened each record and each report retrieved, whether they worked independently, and if applicable, details of automation tools used in the process. | Page 4 |
| Data collection process | 9 | Specify the methods used to collect data from reports, including how many reviewers collected data from each report, whether they worked independently, any processes for obtaining or confirming data from study investigators, and if applicable, details of automation tools used in the process. | Page 4 |
| Data items | 10a | List and define all outcomes for which data were sought. Specify whether all results that were compatible with each outcome domain in each study were sought (e.g. for all measures, time points, analyses), and if not, the methods used to decide which results to collect. | Pages 4 & 5 |
|  | 10b | List and define all other variables for which data were sought (e.g. participant and intervention characteristics, funding sources). Describe any assumptions made about any missing or unclear information. | Page 5 |
| Study risk of bias assessment | 11 | Specify the methods used to assess risk of bias in the included studies, including details of the tool(s) used, how many reviewers assessed each study and whether they worked independently, and if applicable, details of automation tools used in the process. | Page 5 |
| Effect measures | 12 | Specify for each outcome the effect measure(s) (e.g. risk ratio, mean difference) used in the synthesis or presentation of results. | No meta-analysis was conducted. Effect measures were reported narratively based on the descriptions provided in the included studies (e.g., presence or absence of associations or relations). |
| Synthesis methods | 13a | Describe the processes used to decide which studies were eligible for each synthesis (e.g. tabulating the study intervention characteristics and comparing against the planned groups for each synthesis (item #5)). | All six included studies were individually reviewed and summarized. Given the small number of studies and their varied characteristics, no formal grouping or synthesis by intervention type was performed. Each study’s findings were described narratively in the results tables based on the reported outcomes. |
|  | 13b | Describe any methods required to prepare the data for presentation or synthesis, such as handling of missing summary statistics, or data conversions. | No data conversions or imputations were required. Data were extracted as reported in the original studies and presented directly in tables and narrative form without statistical synthesis or transformation |
|  | 13c | Describe any methods used to tabulate or visually display results of individual studies and syntheses. | Page 5 |
|  | 13d | Describe any methods used to synthesize results and provide a rationale for the choice(s). If meta-analysis was performed, describe the model(s), method(s) to identify the presence and extent of statistical heterogeneity, and software package(s) used. | No meta-analysis was performed due to the limited number of studies and their diverse characteristics. Instead, a narrative synthesis approach was used to summarize and interpret the findings, which was deemed appropriate to provide a clear overview without combining heterogeneous data statistically. |
|  | 13e | Describe any methods used to explore possible causes of heterogeneity among study results (e.g. subgroup analysis, meta-regression). | No formal exploration of heterogeneity was conducted because a meta-analysis was not performed and the number of included studies was limited. |
|  | 13f | Describe any sensitivity analyses conducted to assess robustness of the synthesized results. | No sensitivity analyses were conducted due to the narrative synthesis approach and the limited number of studies included. |
| Reporting bias assessment | 14 | Describe any methods used to assess risk of bias due to missing results in a synthesis (arising from reporting biases). | Page 5 |
| Certainty assessment | 15 | Describe any methods used to assess certainty (or confidence) in the body of evidence for an outcome. | No formal assessment of the certainty (or confidence) in the body of evidence was conducted for the outcomes included in this review. |
| **RESULTS** | | |  |
| Study selection | 16a | Describe the results of the search and selection process, from the number of records identified in the search to the number of studies included in the review, ideally using a flow diagram. | Figure 1. PRISMA flowchart of the results from the literature search. |
|  | 16b | Cite studies that might appear to meet the inclusion criteria, but which were excluded, and explain why they were excluded. | Figure 1. PRISMA flowchart of the results from the literature search. |
| Study characteristics | 17 | Cite each included study and present its characteristics. | Table 1. Included study and intervention characteristics |
| Risk of bias in studies | 18 | Present assessments of risk of bias for each included study. | Page 8 |
| Results of individual studies | 19 | For all outcomes, present, for each study: (a) summary statistics for each group (where appropriate) and (b) an effect estimate and its precision (e.g. confidence/credible interval), ideally using structured tables or plots. | Table 2. Outcomes and results summary of the included studies. |
| Results of syntheses | 20a | For each synthesis, briefly summarise the characteristics and risk of bias among contributing studies. | Table 1. Included study and intervention characteristics |
|  | 20b | Present results of all statistical syntheses conducted. If meta-analysis was done, present for each the summary estimate and its precision (e.g. confidence/credible interval) and measures of statistical heterogeneity. If comparing groups, describe the direction of the effect. | No statistical syntheses or meta-analyses were conducted. The review presents a narrative summary of findings, and results are displayed descriptively in summary tables without statistical comparisons or measures of effect. |
|  | 20c | Present results of all investigations of possible causes of heterogeneity among study results. | No investigations of heterogeneity were conducted, as the review did not include statistical synthesis or grouping of study results. |
|  | 20d | Present results of all sensitivity analyses conducted to assess the robustness of the synthesized results. | No sensitivity analyses were conducted, as no statistical synthesis or meta-analysis was performed. |
| Reporting biases | 21 | Present assessments of risk of bias due to missing results (arising from reporting biases) for each synthesis assessed. | No assessment of risk of bias due to missing results was conducted, as no meta-analysis or statistical synthesis was performed. |
| Certainty of evidence | 22 | Present assessments of certainty (or confidence) in the body of evidence for each outcome assessed. | No formal assessment of the certainty or confidence in the body of evidence was conducted for the outcomes included in this review. |
| **DISCUSSION** | | |  |
| Discussion | 23a | Provide a general interpretation of the results in the context of other evidence. | Pages 12-14 |
|  | 23b | Discuss any limitations of the evidence included in the review. | Table 4. Summary of the included study implications and drawbacks. |
|  | 23c | Discuss any limitations of the review processes used. | Page 15 |
|  | 23d | Discuss implications of the results for practice, policy, and future research. | Page 15 |
| **OTHER INFORMATION** | | |  |
| Registration and protocol | 24a | Provide registration information for the review, including register name and registration number, or state that the review was not registered. | This review was not prospectively registered in a public review database. |
|  | 24b | Indicate where the review protocol can be accessed, or state that a protocol was not prepared. | A review protocol was not prepared for this study. |
|  | 24c | Describe and explain any amendments to information provided at registration or in the protocol. | Not applicable, as the review was not registered and no protocol was prepared. |
| Support | 25 | Describe sources of financial or non-financial support for the review, and the role of the funders or sponsors in the review. | This study is part of the project Co-innovation for Digital Rehabilitation in the Global Marketplace, funded by Business Finland (grant 6169/31/2021). The authors declare no involvement of funders or sponsors in the design, conduct, or reporting of the review. |
| Competing interests | 26 | Declare any competing interests of review authors. | The authors declare no competing interests related to this review. |
| Availability of data, code and other materials | 27 | Report which of the following are publicly available and where they can be found: template data collection forms; data extracted from included studies; data used for all analyses; analytic code; any other materials used in the review. | All data extracted from the included studies and summary tables are provided within the manuscript. No template data collection forms, analytic code, or additional materials were used or are publicly available for this review. |

*From:*  Page MJ, McKenzie JE, Bossuyt PM, Boutron I, Hoffmann TC, Mulrow CD, et al. The PRISMA 2020 statement: an updated guideline for reporting systematic reviews. BMJ 2021;372:n71. doi: 10.1136/bmj.n71. This work is licensed under CC BY 4.0. To view a copy of this license, visit <https://creativecommons.org/licenses/by/4.0/>
